# Comparing the effects of problem- and task-based learning on knowledge and clinical decision-making of nursing students concerning the use of transfusion medicine in pediatric nursing: An educational quasi-experimental study in Iran

**DOI:** 10.1016/j.heliyon.2024.e34521

**Published:** 2024-07-15

**Authors:** Soghra Rafie Papkiadeh, Zahra Taheri-Ezbarami, Mahshid Mirzaie Taklimi, Ehsan Kazemnejad Leili, Ali Razaghpoor

**Affiliations:** a Medical Education Research Center, Guilan University of Medical Sciences, Rasht, Iran; b Medical Education Research Center and Shahid Beheshti School of Nursing & Midwifery, Guilan University of Medical Sciences, Rasht, Iran; c Shahid Beheshti School of Nursing & Midwifery, Guilan University of Medical Sciences, Rasht, Iran; d School of Nursing & Midwifery, Qazvin University of Medical Sciences, Qazvin, Iran

**Keywords:** Problem-based learning, Task-based learning, Nursing competence, Transfusion medicine, Nursing students, Pediatric nursing

## Abstract

**Background:**

In the pediatric care field, ensuring safe and effective blood transfusions, promptly identifying adverse reactions, and implementing appropriate interventions are crucial. Therefore, undergraduate nursing curricula need to be structured to meet these professional standards and prepare nursing students, as future team members, to respond to relevant clinical situations. The objective of this study was to investigate how problem- and task-based learning affects knowledge and clinical decision-making of undergraduate nursing students concerning the use of transfusion medicine in pediatric nursing.

**Material and methods:**

This quasi-experimental study involved 82 nursing students recruited from two nursing schools in Iran using convenience sampling. Participants received educational content through either problem- (n = 40) or task-based learning (n = 42) methods. A researcher-made tool, comprising three parts and proven to be valid and reliable, was utilized for data collection. The tool was administered both before and immediately after the intervention. Data were analyzed using Wilcoxon rank-sum, Mann-Whitney U, Spearman's correlation and multivariate analysis of covariance tests via SPSS v16.0. A p-value of <0.05 was considered significant for all tests.

**Results:**

The median post-test knowledge and clinical decision-making scores within problem- and task-based learning groups were 62.68 vs. 74.65 and 53.33 vs. 76.67, respectively. Significant differences were observed between the mean pre- and post-test scores of both variables within both intervention groups (p < 0.05). Multivariate analysis of covariance revealed that task-based learning resulted in significant differences between the two groups in terms of knowledge (F = 87.9 %, p = 0.002, Eta2 = 0.114) and clinical decision-making (F = 99.9 %, p < 0.001, Eta2 = 0.271).

**Conclusions:**

Given the greater effectiveness of task-based learning, nursing schools are advised to utilize this method in undergraduate nursing curricula to ensure the adequacy of the clinical skills acquired by nursing students prior to graduation.

## 1 Introduction

Transfusion medicine encompasses a multidisciplinary science focused on the appropriate utilization and management of blood to prevent or treat diseases [1]. In the pediatric care field, clinical situations necessitating transfusions are deemed urgent, and any delay may result in adverse consequences [2]. The World Health Organization reports that in low-income countries, up to 54 % of blood transfusions are provided to children under the age of 5 [3]. Despite its benefits, transfusion medicine can be associated with serious complications, most of which are primarily caused by mistakes made by healthcare providers at the bedside [2,4,5]. Furthermore, physiological and pathological differences between children and adults pose additional challenges in the field of pediatric care [2,5,6]. Therefore, skilled personnel are required to properly and safely perform this procedure, especially when treating children [5].

Over half of the tasks involved in a transfusion process depend on nurses' clinical skills [7]. However, evidence suggests some incompetencies in this regard. Studies by Abdulredha Abbass et al. and Elbaqary et al. found unsatisfactory levels of knowledge and performance among pediatric nurses [4,8]. Previous research links these incompetencies to inadequate education during nurses' university years and even believes that most receive no education at all [5,9]. Our previous study also revealed similar findings among nursing students in relation to two additional dimensions of nursing competence, referred to as knowledge and clinical decision-making [5]. Furthermore, conventional teaching methods, such as lecture-based learning, have significant drawbacks. Learners often disengage, forget new information quickly, and their individual needs and cognitive skills are neglected. These shortcomings can significantly impact the effectiveness of the teaching [5,10]. Hence, based on the recommendations of medical education experts, it is vital to modify the undergraduate nursing curricula and incorporate innovative teaching techniques. To achieve this, educational strategies must undergo significant changes, and the SPICES model (standing for student-centered, problem-based, integrated, community-based, electives, and systematic) should be taken into consideration. These changes are necessary to ensure the use of the best available evidence and improve the quality of nursing education [5,11,12]. Two innovative teaching methods that can be used in this regard are Problem-Based Learning (PBL) and Task-Based Learning (TBL).

Barrows & Tamblyn believed that learning from problems is inherent to human existence [13]. In PBL, students collaborate in small and self-directed groups, guided by a tutor who acts as a facilitator, director and guide, to learn about a specific subject by finding the solutions to relevant real-world problems [14,15]. In other words, they have the chance to explore, investigate, analyze, synthesize, conduct experiments, and finally, reach conclusions [14]. Numerous studies have examined the impacts of PBL in different areas of the nursing profession. For instance, studies by Razaghpoor et al. regarding transfusion medicine on knowledge and clinical decision-making, Jamshidi et al. regarding patient safety on knowledge, attitude and perception, and Thabet et al. on decision-making skills have demonstrated the effectiveness of this teaching method [2,16,17].

Unlike PBL, which focuses on paper-based problems, learning in TBL revolves round a set of real-world tasks in an authentic setting, addressed and guided by an instructor. In fact, TBL serves as a bridge between theory and practice, wherein students (1) learn about the tasks, (2) develop a deep understanding of the concepts and mechanisms underlying the tasks, (3) apply the knowledge and skills in other fields, and (4) acquire the relevant competencies [18]. Several studies have investigated the effects of TBL among undergraduate and postgraduate medical students. For instance, studies by Sadati et al. on knowledge and practice skill among undergraduate surgical technology students, and Tian et al. on decision-making skills among medical postgraduates have demonstrated the effectiveness of this teaching method [19,20].

### 1.1 Research objectives

After considering all factors, it can be concluded that offering transfusion medicine courses to nursing students and incorporating innovative teaching methods can aid in developing their relevant competencies. Therefore, the objectives of this study were.

1) To compare the knowledge scores of nursing students before and after the intervention within the PBL and TBL groups concerning the use of transfusion medicine in pediatric nursing.
2) To compare the clinical decision-making scores of nursing students before and after the intervention within the PBL and TBL groups concerning the use of transfusion medicine in pediatric nursing.
3) To compare the knowledge and clinical decision-making scores of nursing students before and after the intervention between the PBL and TBL groups concerning the use of transfusion medicine in pediatric nursing.

### 1.2 Research hypothesis

The utilization of TBL to instruct nursing students on the use of transfusion medicine in pediatric nursing proves to be more effective than PBL in enhancing their knowledge and clinical decision-making.

## 2 Materials and methods

### 2.1 Study design

This quasi-experimental study employed a pre- and post-test design, and was carried out in two nursing schools, one with a PBL-based intervention and the other with a TBL-based intervention, located at Guilan Province, Iran, from May to June 2019.

### 2.2 Participants

**Fig. 1:**
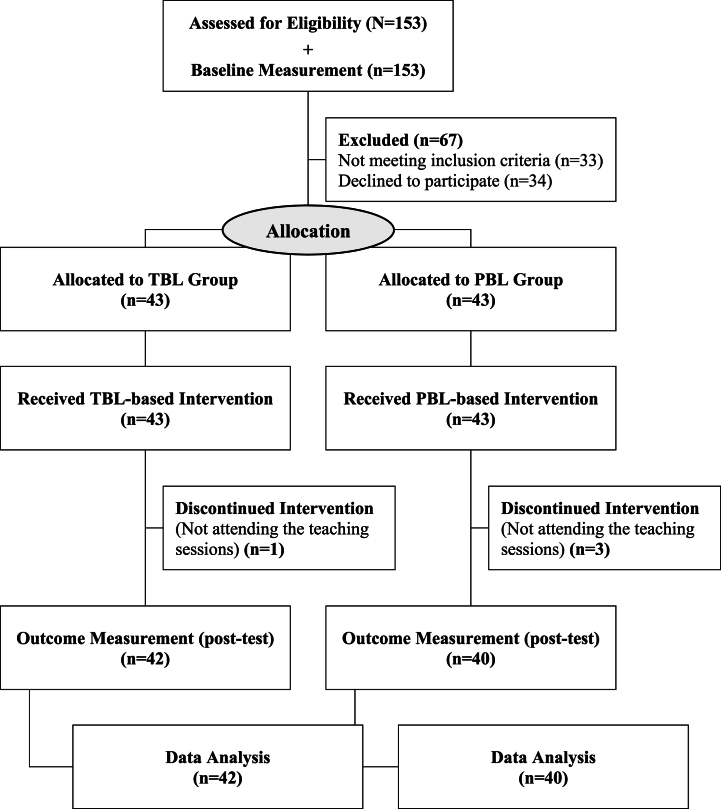
Flow of study procedure.

The study population included 2nd- and 3rd-year undergraduate nursing students who passed the adult/gerontological nursing II and the pediatric nursing courses, and had not previously worked as staff in hospitals or the Iranian Blood Transfusion Organization (IBTO). Students who missed the teaching sessions, incompletely filled out the questionnaires or were unwilling to continue cooperation were excluded. Due to single blinding, participants were selected from two schools within the same university. All eligible students in both schools were first asked to fill out the questionnaires. Following the determination of a cut-off point, participants were then recruited from those who scored <75 % of the total score using convenience sampling. The sample size was calculated based on Vakani et al.'s study [21], with a confidence interval of 95 %, a test power of 90 and a 10 % drop out rate, resulting in a sample size of 43 in each group (86 in total) (Fig. 1).

$$n=\frac{\left({\left(z_{1-\frac{\alpha \;}{2}}+z_{1-\beta }\right)}^{2}\;\left({SD_{1}}^{2}+{SD_{2}}^{2}\right)\right)}{d^{2}}$$

$$1–\alpha =0/95;\;z_{1-\frac{\alpha \;}{2}}=z_{0/975}=1/96$$

$$1–\beta =0/90;\;z_{1-\beta }=z_{0/90}=1/28$$

### 2.3 Measurements

A researcher-made tool comprising three parts was utilized for data collection. The tool was administered both before and immediately after the intervention [20]. The tool was developed by reviewing relevant studies and questionnaires, and is both valid and reliable [5].

#### 2.3.1 Demographics

This section consisted of participants’ age, gender, student work experience, and blood transfusion experience.

#### 2.3.2 Knowledge

This section evaluated the knowledge concerning the use of transfusion medicine in pediatric nursing. The questionnaire included 71 items, assessing knowledge in 6 domains of “blood storage (10 items)", “blood use (6 items)", “blood transfusion reactions (8 items)", “nursing interventions before the transfusion (21 items)", “nursing interventions during the transfusion (16 items)" and “nursing interventions after the transfusion (10 items)". Participants provided their responses using a 3-point Likert scale (True/False/Don't Know), where 1 indicated a “correct” response, and 0 indicated an “incorrect or don't know” response. Total score ranged between 0 and 71.

#### 2.3.3 Clinical decision-making

This section evaluated the clinical decision-making concerning the use of transfusion medicine in pediatric nursing. The questionnaire included 15 multiple-choice scenario questions. Correct answers were assigned a score of 1, and incorrect answers were assigned a score of 0. Total score ranged between 0 and 15.

The linear transformation technique was employed to normalize the raw scores on both questionnaires, with the total score being adjusted to fall within the range of 0–100 [22].

For qualitative content validity, 14 experts (pediatric hematologists/oncologists, physicians in charge of hemovigilance at a pediatric hospital, nurses working in pediatric thalassemia and hematology wards, faculty members of the school of nursing & midwifery, and physicians working in the IBTO) were selected purposively. They were then requested to comment on the items, and any necessary changes were made based on their feedback. To assess quantitative content validity, Lawshe's content validity ratio (CVR) and Waltz and Bausell's content validity index (CVI) were employed. Items with a CVR score greater than 0.57 [23] and a CVI score greater than 0.85 [24] were kept. We also calculated the S-CVI, which yielded a score of 0.90 [24]. To assess reliability, 20 students were selected. Internal consistency reliability was evaluated using the Kuder-Richardson 20, while test-retest reliability was measured through intraclass correlation coefficient and parallel forms reliability.

### 2.4 Data collection

Initially, the educational content was compiled after reviewing related scientific sources and surveying related errors reported in thalassemia, hematology and internal wards. This content covered the following topics: definition of transfusion medicine, blood products, nursing interventions before, during, and after transfusions, as well as different types of transfusion reactions and their management. Next, the content underwent scientific validation by a team comprising pediatric hematologists/oncologists, physicians from the IBTO, nurses from a pediatric hospital and faculty members from the pediatric nursing department. Following baseline measurement, participants in both intervention groups received similar educational content delivered via either PBL or TBL methods. It should be made clear that the same data collection tool was utilized. The entire process was carried out within a span of 2 months.

#### 2.4.1 PBL group

For participants in this group, a number of scenarios were first crafted based on real-world problems concerning the use of transfusion medicine in pediatric nursing. An example of these scenarios is as follows: “The patient is a 10-year-old boy who became pale and lethargic after being exposed to the bean garden. According to the mother, the child's urine is dark, and he exhibits symptoms of nausea and vomiting. Based on the lab results, the child's hemoglobin is 6.5 mg/dL” The intervention involved two in-person sessions (Table 1) that lasted a total of 4 h. In the first session lasting 1 h, the researchers presented the course plan and briefly covered the educational content through lecture. Students were then randomly divided into three groups, each consisting of 14–15 individuals. In the second session lasting 3 h, the researchers served as facilitators, working alongside the students.

**Table 1:** Steps of PBL-based intervention (n = 40).

Sessions	duration	what to do?	details
1st Session	1 h	Preliminary Preparations	1. A number of scenarios were crafted based on the real-world problems concerning the use of transfusion medicine in pediatric nursing. 2. The course plan and educational content were provided to students. 3. Students were randomly divided into three groups, each consisting of 14–15 individuals. Once students were in groups, each member was assigned a responsibility and a representative was chosen for each group by the members.
2nd Session	Phase 1 (2 h)	Learning Operations and Activities	Several scenarios were provided to each group so that members could identify the necessary information and key problems, and discuss the findings within their groups to find solutions.
	Phase 2 (1 h)	Group Discussion and Summarization	1. Groups convened in one location, and the findings within each group were shared with other groups and summarized. 2. Researchers addressed groups' strengths and weaknesses, resolving students' problems and ambiguities.

#### 2.4.2 TBL group

For participants in this group, a list of tasks that all nurses must perform concerning the use of transfusion medicine in pediatric wards, including thalassemia, hematology and internal wards, was compiled. An example of these tasks is as follows: “What education should be given to a child diagnosed with Favism?” The intervention involved two in-person sessions (Table 2) that lasted a total of 6 h. In the first session lasting 1 h, the researchers presented the course plan and briefly covered the educational content through lecture. Students were then randomly divided into seven groups, each consisting of 6–7 individuals. During this session, a representative was also chosen for each group. In the second session lasting 5 h, the intervention was conducted through 4 phases, as illustrated in Table 2.

**Table 2:** Steps of TBL-based intervention (n = 42).

Sessions	duration	what to do?	details
1st Session	1 h	Preliminary Preparations	1. A list of tasks that all nurses must perform concerning the use of transfusion medicine in pediatric wards was made. 2. The course plan and educational content were provided to students. 3. Students were then randomly divided into seven groups, each consisting of 6–7 individuals. Once students were in groups, each member was assigned a responsibility and a representative was chosen for each group by the members.
2nd Session	Phase 1 (2 h)	Learning Activities	Under the supervision of the representatives, members researched the necessary information from various sources according to the course plan and nurses' tasks. They then discussed the key points within their groups.
	Phase 2 (1 h)	Learning Operations	A scenario was provided to each group so that members could identify the necessary information regarding the diseases or nurses' tasks, and determine the correct responses to the questions given to them.
	Phase 3 (1 h)	Group Discussion	1. Groups convened in one location, and the findings within each group were presented and shared with other groups by the representatives. 2. All learnings were briefly reviewed by one of the seven representatives.
	Phase 4 (1 h)	Summarization	Researchers addressed groups' strengths and weaknesses, resolving students' problems and ambiguities.

### Ethical approval

This study was carried out after receiving ethics approval (IR.GUMS.REC.1397.509) from the Ethics Committee on March 16th, 2019, an official introduction letter from the Vice-Chancellor of Research and Technology at Guilan University of Medical Sciences, and required permits from the educational departments in both schools. All participants provided written informed consent after receiving detailed explanations on the study's nature, objectives and methodology. They were also informed that participation was voluntary, and they could withdraw from the study at any point. Furthermore, they were assured that their information would remain confidential throughout all stages. A gift (a notebook and a pen) was also provided to each participant as a gesture of appreciation for their cooperation.

### 2.5 Data analysis

Data were analyzed using SPSS for Windows, version 16.0. Descriptive statistics were used to describe quantitative and qualitative variables. Normality was tested using the Kolmogorov-Smirnov and Shapiro-Wilk tests, revealing that knowledge and clinical decision-making did not follow a normal distribution (p < 0.05). Consequently, non-parametric tests were employed. Wilcoxon rank-sum and Mann-Whitney U tests were utilized, respectively, to compare the mean pre- and post-test knowledge and clinical decision-making scores within and between the intervention groups. Spearman's correlation coefficient was utilized to examine the correlation between the mean pre- and post-test knowledge and clinical decision-making scores. To assess the impact of both methods on the mean post-test knowledge and clinical decision-making scores, a multivariate analysis of covariance (MANCOVA) was conducted. A p-value of <0.05 was considered significant for all tests.

## 3 Results

### 3.1 Demographic characteristics

Eighty-two nursing students, comprising 40 in PBL group and 42 in TBL group, participated in this study. Demographic characteristics showed no significant differences between the two groups, indicating they were homogeneous (p < 0.05) (Table 3).

**Table 3:** Students’ demographic characteristics in both groups.

variables		participants (n = 82)				p-value
		PBL group (n = 40)		TBL group (n = 42)		
		Freq.	%	Freq.	%	
age (min = 19, max = 40)	<21 years	24	60.00	25	59.52	0.965^a^
	21≤ years	16	40.00	17	40.48	
M±SD (in years)		21.58 ± 2.54		21.98 ± 3.11		
gender	female	24	60.00	21	50.00	0.363^a^
	male	16	40.00	21	50.00	
student work experience	Yes	6	15.00	5	11.90	0.681^a^
	No	34	85.00	37	88.10	
blood transfusion experience	Yes	3	7.50	4	9.52	0.999^b^
	No	37	92.50	38	90.48	

M: mean/SD: Standard Deviation.

a Chi-square test/

b Fisher's exact test.

### 3.2 Knowledge and clinical decision-making

Table 4 indicates no significant differences in the mean pre-test knowledge (p = 0.365) and clinical decision-making (p = 0.981) scores between the PBL and TBL groups. However, the mean post-test knowledge (p = 0.005) and clinical decision-making (p < 0.001) scores were both significantly lower in PBL group compared to TBL group. Additionally, significant differences were found between the mean pre- and post-test knowledge and clinical decision-making scores within both the PBL (p < 0.001) and TBL (p < 0.001) groups.

**Table 4:** Within- and between-group comparison of the pre- and post-test knowledge and clinical decision-making scores.

variables	groups	before intervention			after intervention			p-value
		M	SD	median (IQR)	M	SD	median (IQR)	
knowledge	PBL (n = 40)	45.46	19.54	50.70 (38.73)	61.20	16.66	62.68 (16.55)	0.001^a^
	TBL (n = 42)	40.38	22.41	47.18 (40.14)	69.78	17.53	74.65 (20.07)	0.001^a^
**p-value**		0.365^b^			0.005^b^			
clinical decision-making	PBL (n = 40)	26.83	18.46	23.33 (26.67)	55.67	16.37	53.33 (20.00)	<0.001^a^
	TBL (n = 42)	27.62	21.37	26.67 (40.00)	72.54	15.74	76.67 (15.00)	<0.001^a^
**p-value**		0.981^b^			<0.001^b^			

M: Mean/SD: Standard Deviation/IQR (Q3-Q1): Interquartile Range.

a Wilcoxon rank-sum test/

b Mann-Whitney U tests.

### 3.3 Correlations between knowledge and clinical decision-making

Table 5 illustrates significant and direct correlations within PBL group between the mean pre- and post-test knowledge scores (p = 0.001, r = 0.503), the mean pre- and post-test clinical decision-making scores (p < 0.001, r = 0.575), and the mean pre-test knowledge score and the mean post-test clinical decision-making score (p = 0.001, r = 0.495). However, in TBL group, a significant and positive correlation was only found between the mean pre- and post-test knowledge scores (p = 0.021, r = 0.355).

**Table 5:** Correlations between the mean pre- and post-test knowledge and clinical decision-making scores in PBL and TBL groups.

groups	variables	values	post-test score of knowledge	post-test score of clinical decision-making
PBL (n = 40)	pre-test score of knowledge	r^a^	0.503	0.495
		p-value	0.001	0.001
	pre-test score of clinical decision-making	r^a^	0.158	0.575
		p-value	0.330	<0.001
TBL (n = 42)	pre-test score of knowledge	r^a^	0.355	0.286
		p-value	0.021	0.066
	pre-test score of clinical decision-making	r^a^	0.193	0.239
		p-value	0.221	0.128

a Spearman's rank correlation coefficient.

### 3.4 Effectiveness of PBL- and TBL-based interventions

Based on the abovementioned results, controlling the mean pre-test knowledge and clinical decision-making scores is necessary to investigate the effects of the interventions on their mean post-test scores. The MANCOVA test was used to determine the significance of these changes (Table 6). Concerning pretest scores, TBL resulted in significant differences between both groups in terms of knowledge (F = 87.9 %, p = 0.002, Eta2 = 0.114) and clinical decision-making (F = 99.9 %, p < 0.001, Eta2 = 0.271). Additionally, the mean pre-test clinical decision-making scores had no effect on the mean post-test knowledge and clinical decision-making scores (p > 0.05). However, the mean pre-test knowledge scores significantly influenced both the mean post-test knowledge (p < 0.001) and clinical decision-making (p = 0.048) scores.

**Table 6:** Results of MANCOVA test.

source	dependent variable	type III sum of squares	df	mean square	f	Sig.	partial eta squared	observed power
pre-test score of knowledge	post-test score of knowledge	3940.4	1	3940.4	17.1	<0.001	0.180	0.983
	post-test score of clinical decision-making	874.8	1	874.8	4.0	0.048	0.049	0.511
pre-test score of clinical decision-making	post-test score of knowledge	68.1	1	68.1	0.3	0.588	0.004	0.084
	post-test score of clinical decision-making	0.568	1	0.568	2.6	0.109	0.033	0.360
educational interventions	post-test score of knowledge	2311.7	1	2311.7	10.0	0.002	0.114	0.879
	post-test score of clinical decision-making	6272.5	1	6272.5	29.0	<0.001	0.271	0.999

## 4 Discussion

This study investigated how PBL and TBL affects knowledge and clinical decision-making of undergraduate nursing students concerning the use of transfusion medicine in pediatric nursing.

Results demonstrated that PBL significantly enhanced both variables. Consistent with these results, a study by Razaghpoor et al. highlighted the efficacy of PBL in enhancing nursing students' competence in applying transfusion medicine in pediatric nursing [2].

These positive outcomes can be attributed to the characteristics of PBL, which include promoting active, group-based, self-directed, and evidence-based learning. PBL also motivates learners and helps them develop the necessary competencies to solve real-world problems [2,25]. Regarding other fields of nursing education, results showed similar findings. For instance, Jamshidi et al. demonstrated that PBL significantly influences nursing students’ knowledge, attitude, and perception regarding patient safety when compared to traditional teaching methods [16]. In another study, Thabet et al. showed that PBL plays a crucial role in enhancing nursing students' decision-making skills [17]. However, results of limited studies are inconsistent with this finding. Solomon, for instance, demonstrated that PBL has minimal influence on the immediate retention of knowledge among nursing students in comparison to lecture-based learning. They believed that since lecture-based learning encourages surface learning, students will better be able to recall what was covered in the sessions; meanwhile, PBL promotes in-depth comprehension, allowing students to concentrate on finding significance and implementing knowledge in real-world clinical scenarios rather than simply memorizing information [26]. Therefore, it seems that PBL, as a pedagogy, has great potential concerning the use of transfusion medicine in pediatric nursing.

Similar to PBL, results showed that TBL is also significantly effective on both variables. It seems that designing education based on real-world tasks, in a real-world setting, and in collaboration with a group can be useful in enhancing nursing students' knowledge and clinical decision-making. Although no study was found on the impact of TBL on learning outcomes related to transfusion medicine in nursing, results of previous studies in other areas of medical education are in line with this finding. In two studies comparing the effects of TBL and a conventional teaching method on undergraduate surgical technology students’ knowledge, practical skills and speed of action, Sadati et al. and Hannani et al. stated that TBL is a more efficient method in the clinical education compared to conventional teaching methods [19,27]. In another study, Tian et al. investigated the effects of a modified TBL model on problem-solving abilities among postgraduate medical students and showed that TBL helps them develop the necessary competencies by promoting learning motivation, developing self-learning abilities, enhancing learning efficiency, and developing problem-solving, collaboration and communication skills [20]. Since skilled personnel are required to properly and safely administer blood transfusions to patients, especially when treating children, and the best time and place for education is during university years and at the bedside under the supervision of an instructor, application of TBL can be a practical and appropriate method.

In addition to improving teaching and learning, student-centered teaching methods deepen the learning process, increase leaners' satisfaction with learning, and promote their abilities [28]. Regarding the comparison of the effects of the two student-centered teaching methods, results showed that TBL is a more efficient method compared to PBL in improving both nursing students' knowledge and clinical decision-making concerning the use of transfusion medicine in pediatric nursing. Within the field of medical education, only one similar study was found. In line with this study, Vakani et al. showed that both TBL and problem-oriented lecture methods are equally effective in improving general practitioners' perceptions and clinical reasoning skills; however, TBL was more effective [21]. In one qualitative study, Ozan et al. determined students' perceptions on the efficacy of the school's educational programs and showed that TBL increases their self-awareness and self-esteem and strengthens their collaboration and communication skills. The method also makes the learning process more pleasant, encourages learners to use theory in real-world situations, and improves adaptability in the workplace [29]. Nonetheless, since each stage of knowledge acquisition requires a specific type of learning approach or teaching method, and each teaching method is associated with certain limitations and challenges, combining them may be useful to make the process easier for students to achieve their goals. In this regard, Takahashi also believes that since learners in PBL may confront with some challenges in practically and effectively implementing their learnings, combining it with TBL, which provides the opportunists to actually apply the acquired knowledge and skills in PBL, can eliminate these disadvantages [30].

### 4.1 Strengths and limitations

To our knowledge, this study is among the first of its kind to utilize real-world nursing scenarios, particularly focusing on nurses' tasks and problems regarding transfusion medicine, to enhance nursing students' clinical skills. This was accomplished through the incorporation of TBL and PBL methods. Additionally, the educational content underwent review by a specialized team, and its practical applications in pediatric nursing were employed to educate students, where there was previously no formal education program; namely, the integration of pediatric transfusion medicine into nursing. Consequently, incorporating this content into the nursing curriculum can lead to improved performance among graduate nurses and is anticipated to enhance patient safety and care quality in the future. The participation of nursing students in group sessions strengthened their problem-solving skills. Another strength of the study lies in the tools utilized, developed with input from experienced professionals in the field of transfusion medicine, which were employed in a cross-sectional study [5] before this one. This study has potential limitations, as follows: (1) the influence of participants' psychological state on their responses to the data collection tool, which was beyond the researchers’ control; (2) Brainstorming while completing the data collection tool, for which researchers tried to simulate the conditions of a real exam; (3) Utilizing a self-report data collection tool; (4) Conducting the intervention in two groups at different times; and (5) Selecting the intervention groups from two different schools, which was inevitable due to single blinding.

## 5 Conclusion

The results of this study demonstrated that despite the significant effectiveness of both methods on improving students’ learning outcomes, TBL was more efficient. These results can be beneficial in nursing education, clinical nursing and nursing research. For nursing education, it is recommended to nursing schools to apply TBL in undergraduate nursing curriculums in order to ensure the adequacy of the clinical skills acquired by nursing students prior to graduation. In addition, the combination of PBL and TBL methods is recommended in the field of transfusion medicine and other fields of nursing education. For clinical nursing, both methods as well as the educational content can be used to design and conduct effective continuing education programs for nursing professionals. For nursing research, the methods and results can be a guide for future studies regarding application and comparison of active teaching methods in nursing education field, especially in the field of pediatric nursing.

### Funding

No specific grant from a public, private, or nonprofit funding organization was used to support this research.

### Data availability statement

Due to the ethics of this research, the data was not shared in a publicly available repository. Data used to support the findings of this study are available from the corresponding author upon request.

## CRediT authorship contribution statement

**Soghra Rafie Papkiadeh:** Writing – review & editing, Writing – original draft, Validation, Supervision, Project administration, Methodology, Investigation, Formal analysis, Data curation, Conceptualization. **Zahra Taheri-Ezbarami:** Writing – review & editing, Writing – original draft, Visualization, Validation, Supervision, Software, Resources, Project administration, Methodology, Investigation, Formal analysis, Data curation, Conceptualization. **Mahshid Mirzaie Taklimi:** Writing – review & editing, Writing – original draft, Validation, Supervision. **Ehsan Kazemnejad Leili:** Writing – review & editing, Writing – original draft, Validation, Software, Methodology, Investigation, Formal analysis, Data curation. **Ali Razaghpoor:** Writing – review & editing, Writing – original draft, Resources, Formal analysis.

## Declaration of competing interest

The authors declare that they have no known competing financial interests or personal relationships that could have appeared to influence the work reported in this paper.
