# Supplementary material for: Comparing the effects of problem- and task-based learning on knowledge and clinical decision-making of nursing students concerning the use of transfusion medicine in pediatric nursing: An educational quasi-experimental study in Iran
Source: Heliyon. 2024 Jul 15;10(14):e34521. doi: 10.1016/j.heliyon.2024.e34521 (PMC11330116; doi:10.1016/j.heliyon.2024.e34521)
Supplement: Multimedia component 1 [file mmc1.docx]

Dear nursing student,

This tool was developed to collect information for a study aiming to *compare the effects of problem- and task-based learning on nursing students’ knowledge and clinical decision-making regarding application of transfusion medicine in pediatric nursing*.

The tool consists of three parts, as follows:

1. Demographics
2. Knowledge
3. Clinical Decision-Making
4. ***Demographics***

1. **Age:** ……………… (in years)

2. **Gender:** female 🗆 male 🗆

3. **Do you have work experience as a student?** Yes 🗆 No 🗆

4. **Have you ever administered blood products to a patient?** Yes 🗆 No 🗆

1. ***Knowledge***

| **blood storage** | | | | |
| --- | --- | --- | --- | --- |
| **items** | | **true** | **false** | **don’t know** |
| **1** | For the storage of whole blood and packed red blood cells, a temperature range of 1 to 6 degrees Celsius, and for their transportation, a range of 1 to 10 degrees Celsius is suitable. |  |  |  |
| **2** | The storage duration of packed red blood cells with CPDA-1 anticoagulant is 45 days. |  |  |  |
| **3** | Platelets can be stored for up to 3 days at a temperature range of 1 to 6 degrees Celsius. |  |  |  |
| **4** | To prevent the deterioration of platelets, platelet bags should be stored at a temperature range of 20 to 25 degrees Celsius, along with gentle shaking. |  |  |  |
| **5** | Fresh frozen plasma must be thawed in water at a temperature range of 30 to 37 degrees Celsius before transfusion. |  |  |  |
| **6** | Temperatures higher than 37 degrees Celsius during the thawing process of fresh frozen plasma can deteriorate coagulation factors and proteins. |  |  |  |
| **7** | As soon as fresh frozen plasma is thawed, it should be stored in a refrigerator at a temperature range of 2 to 6 degrees Celsius. |  |  |  |
| **8** | Fresh frozen plasma can still be used up to 48 hours after thawing, if stored in a refrigerator. |  |  |  |
| **9** | After thawing, cryoprecipitate can be stored for up to 6 hours at a temperature range of 20 to 24 degrees Celsius. |  |  |  |
| **10** | Cryoprecipitate kept at room temperature can be used up to 24 hours after thawing. |  |  |  |

| **blood use** | | | | |
| --- | --- | --- | --- | --- |
| **items** | | **true** | **false** | **don’t know** |
| **1** | When a plasma transfusion is required, there is no need for a cross-match test. |  |  |  |
| **2** | The decision about blood transfusions in children should not rely solely on hemoglobin levels; a careful evaluation of the clinical condition is also necessary. |  |  |  |
| **3** | For plasma, the ABO blood type compatibility between organ donors and recipients is required. |  |  |  |
| **4** | The unit of packed red blood cells that is being transfused must be compatible with the recipient's plasma ABO antibodies. |  |  |  |
| **5** | If the blood product needs to be warmed, water cannot be used. |  |  |  |
| **6** | The use of leukoreduced red blood cells can be useful in reducing febrile nonhemolytic transfusion reactions. |  |  |  |

| **blood transfusion reactions** | | | | |
| --- | --- | --- | --- | --- |
| **items** | | **true** | **false** | **don’t know** |
| **1** | Fever during a blood transfusion can be caused by bacterial contamination. |  |  |  |
| **2** | Fever during a blood transfusion can be caused by hemolytic reaction. |  |  |  |
| **3** | Fever during a blood transfusion can be caused by febrile nonhemolytic transfusion reactions. |  |  |  |
| **4** | Fever during a blood transfusion can be caused due to transfusion-related acute lung injury. |  |  |  |
| **5** | Urticaria can be a symptom of febrile nonhemolytic transfusion reactions. |  |  |  |
| **6** | In transfusion-related acute lung injury, symptoms usually appear within the first 6 hours after the transfusion. |  |  |  |
| **7** | Aspirin cannot be administered for febrile nonhemolytic transfusion reactions |  |  |  |
| **8** | A febrile reaction is defined as an increase of ≥1 degree Celsius during a blood transfusion and/or within the first two hours after the transfusion. |  |  |  |

| **nursing interventions before the transfusion** | | | | |
| --- | --- | --- | --- | --- |
| **items** | | **true** | **false** | **don’t know** |
| **1** | The physician's written order regarding a blood transfusion must be verified in the patient's records. |  |  |  |
| **2** | The patient's identity must be verified against his/her records. |  |  |  |
| **3** | The blood transfusion consent form must be signed by the patient's companion. |  |  |  |
| **4** | The blood group labeled on the blood bag must be verified. |  |  |  |
| **5** | The Rh group labeled on the blood bag must be verified. |  |  |  |
| **6** | A cross-match test must be conducted. |  |  |  |
| **7** | The serial numbers of blood bags must be checked. |  |  |  |
| **8** | The expiration dates of blood bags must be verified. |  |  |  |
| **9** | The integrity of the blood bag must be checked for the absence of blood clots, bubbles, and overall appearance. |  |  |  |
| **10** | The cord of the bag must be checked for the absence of a metal clip. |  |  |  |
| **11** | The blood product and its volume must be verified. |  |  |  |
| **12** | The blood transfusion set must be checked to ensure proper movement of the product. |  |  |  |
| **13** | The procedure must be explained to the patients or their companions. |  |  |  |
| **14** | Patients must be informed about any unusual symptoms, such as dizziness, itching, rash, urticaria, chills, flushing, pain, and shortness of breath. |  |  |  |
| **15** | Nurses must wash their hands before the transfusions. |  |  |  |
| **16** | Nurses must wear sterile gloves before the transfusions. |  |  |  |
| **17** | Nurses must prepare the necessary equipment before the transfusions. |  |  |  |
| **18** | Vital signs must be monitored and documented in a special chart before the transfusions. |  |  |  |
| **19** | Any veins may be used for transfusions. |  |  |  |
| **20** | The injection site must be disinfected with gauze swabs, rubbing periodically from the inside to the outside. |  |  |  |
| **21** | An appropriate intravenous line must be established for the transfusions. |  |  |  |

| **nursing interventions during the transfusion** | | | | |
| --- | --- | --- | --- | --- |
| **items** | | **true** | **false** | **don’t know** |
| **1** | Vital signs, including temperature, pulse rate, blood pressure, and respiratory rate, must be monitored immediately, at 15 minutes, 30 minutes, 1 hour, 2 hours, 3 hours after the transfusion begins. |  |  |  |
| **2** | Fluid intake through intravenous and oral routes must be monitored. |  |  |  |
| **3** | The most important intervention during an acute hemolytic reaction is to maintain blood pressure. |  |  |  |
| **4** | The most important intervention during an acute hemolytic reaction is to maintain renal function. |  |  |  |
| **5** | The most important intervention during an acute hemolytic reaction is to prevent disseminated intravascular coagulation. |  |  |  |
| **6** | The urine output must be monitored. |  |  |  |
| **7** | Any unusual symptom must be monitored. |  |  |  |
| **8** | Transfusion of ABO incompatible packed red blood cells may cause an acute hemolytic reaction. |  |  |  |
| **9** | In case of anaphylactic transfusion reactions, elevating the legs may be helpful. |  |  |  |
| **10** | In case of anaphylactic transfusion reactions, an epinephrine can be administered. |  |  |  |
| **11** | In case of anaphylactic transfusion reactions, administering antihistamines may be helpful. |  |  |  |
| **12** | During blood exchange, infants should not be fed orally. |  |  |  |
| **13** | During blood exchange, resuscitation equipment and airway maintenance must be available. |  |  |  |
| **14** | During blood exchange in infants, the umbilical catheter must be inserted using sterile technique. |  |  |  |
| **15** | During blood exchange in infants, the venous catheter must be inserted using sterile technique. |  |  |  |
| **16** | For blood exchange in infants, blood must be administered using a venous catheter. |  |  |  |

| **nursing interventions after the transfusion** | | | | |
| --- | --- | --- | --- | --- |
| **items** | | **true** | **false** | **don’t know** |
| **1** | Nurses must remove gloves after properly disposing of contaminated items. |  |  |  |
| **2** | Nurses must wash their hands after the transfusion. |  |  |  |
| **3** | Vital signs, including temperature, pulse rate, blood pressure and respiratory rate, must be monitored and documented immediately after the transfusion finishes. |  |  |  |
| **4** | The blood transfusion monitoring form must be completed at the end of the transfusions. |  |  |  |
| **5** | The amount of blood transfused must be documented in the patient's records. |  |  |  |
| **6** | The time at which the transfusion is finished must be documented in the patient's records. |  |  |  |
| **7** | The occurrence of any unusual symptoms must be documented in the patient's records. |  |  |  |
| **8** | Patients' vital signs must be checked 4 hours after the transfusion finishes. |  |  |  |
| **9** | At least 4 hours after the blood exchange, infants must not be fed orally. |  |  |  |
| **10** | In case of an incompatible reaction, the remaining blood must be sent to the hospital's blood bank. |  |  |  |

1. ***Clinical Decision-Making***

1. Two units of blood are reserved for a patient. Before initiating the transfusion, what important steps should a nurse take to properly identify the patient?

A. If possible, request the patient to say his/her name.

B. Simply cross-reference the patient's name with the application form.

C. Ensure that the patient's identification details align with those on the blood bag, patient's wristband, and application form.

D. Verify the information on the patient's wristband against that on the blood bag.

2. Two units of blood are ordered for a patient with thalassemia. The nurse administered the first unit to the patient at 10 am after preparing it. Due to its concentration, the transfusion extended until 1 p.m. What is the maximum time the nurse has to administer the first unit?

A. The first unit can be continued until 2 p.m.

B. He/she should discontinue the first unit, and administer the second unit.

C. The first unit can be continued until 5 p.m.

D. He/she should not commence the transfusion of the second unit until the completion of the first one.

3. Two units of blood are ordered for a pediatric patient with a heart disease. How should these units be administered to this patient?

A. The transfusion rate should be adjusted similar to other patients.

B. There is no necessity to adjust the transfusion rate.

C. The blood should be administered slowly.

D. The transfusion rate should be adjusted following the physician's order.

4. Two units of blood are ordered for a patient. During the preparation, the nurse notices that the blood is concentrated and must be diluted. Which of the following solutions can the nurse use to dilute the blood?

A. D/W 5%

B. N/S 0.9%

C. Ringer's Lactate

D. Furosemide 20 mg

5. A 9-year-old girl diagnosed with favism requires two units of blood. To monitor the short-term complications, how long should the nurse observe her after administering the blood?

A. within the first 4 hours

B. within the first 10 to 15 minutes

C. during the transfusion

D. during the shift

6. Twenty minutes after the blood transfusion begins, the patient experiences urticaria, itching all over the body, nausea, vomiting, low blood pressure, and chest pain. What complication is the patient facing?

A. mild allergic reaction

B. hemolytic reaction

C. acute hemolytic reaction

D. transfusion-related acute lung injury

7. The nurse identifies an acute hemolytic reaction in the patient during a blood transfusion. What should be the nurse's initial action?

A. Stopping the transfusion and informing the physician

B. Notifying the blood bank

C. Keeping the vein open with N/S 0.9%

D. Notifying the supervisor

8. What actions would a nurse take if a patient experiences urticaria and itching during a blood transfusion, and the symptoms alleviate after the physician prescribes an antihistamine?

A. She should continue the transfusion after a slightly delay.

B. She should stop the transfusion.

C. She should continue the transfusion promptly.

D. Following the administration of a solution to boost blood pressure, she should continue the transfusion.

9. After a pediatric patient experienced urticaria and itching for the second time following blood transfusion with a leukocyte filter, which type of blood would you choose for transfusion among the provided options?

A. washed red blood cells

B. whole blood

C. leukoreduced red blood cells

D. washed whole blood

10. What would you do if one unit of a patient's blood bag out of two exhibits clotting and/or abnormal turbidity?

A. We should initiate the transfusion of the next unit.

B. We should return both units to the blood bank.

C. We should dilute the blood bag with a solution to alleviate its turbidity.

D. We should return only the bag containing the clot to the blood bank.

11. Two units of blood are prepared for a patient. Upon inspection, the nurse observes that on the application form, the serial number of one unit is distorted, and the serial number of the other unit is repeated twice. What action should be taken in this situation?

A. The nurse must obtain the serial number of the distorted bag from the blood bank over the phone, record it, and proceed with the administration.

B. Both bags must be returned to the blood bank to correct the serial numbers.

C. The transfusion of one unit must commence.

D. Only the bag with the distorted serial number must be returned to the blood bank.

12. Five units of platelets are reserved for a 12-year-old boy with platelets count of 10000. After bringing the platelets to the ward, the nurse notices visible streaks in one of the bags. What should the nurse do in this situation?

A. He/she should transfuse the platelets.

B. He/she should not transfuse the platelets.

C. He/she should return the platelets to the blood bank.

D. After gently shaking, he/she should transfuse the platelets.

13. A 10-year-old boy, possibly diagnosed with favism, was hospitalized and is now being discharged. The patient's mother inquires with the ward nurse about re-examining G6PD test to confirm the diagnosis. What would be the best response the nurse could provide to the patient's mother?

A. four days after the discharge

B. one week after the discharge

C. twenty-one days after the discharge

D. four week after the discharge

14. Which fluid would be appropriate for a pediatric patient diagnosed with favism, as prescribed by the physician?

A. cherry juice

B. pomegranate juice

C. cherry and pomegranate juice

D. sweet and colorless juices

15. While performing her daily tasks, a nurse, who has recently transferred to the blood ward, notices the physician's order to monitor the urine of a pediatric patient with favism every 4 hours. Curious about the rationale behind this test, the nurse approaches the ward head nurse for an explanation. What would be the head nurse's response?

A. To check urine for protein

B. To check urine for electrolytes

C. To check urine for electrolytes and protein

D. To check urine for erythrocyte lysis
